# Photobiomodulation Controls Keratinocytes Inflammatory Response through Nrf2 and Reduces Langerhans Cells Activation

**DOI:** 10.3390/antiox12030766

**Published:** 2023-03-21

**Authors:** Sara Salman, Cyprien Guermonprez, Laurent Peno-Mazzarino, Elian Lati, Audrey Rousseaud, Lieve Declercq, Saadia Kerdine-Römer

**Affiliations:** 1 Inserm, Inflammation Microbiome Immunosurveillance, Université Paris-Saclay, 91400 Orsay, France; 2 Lightinderm, Pépinière Paris Santé Cochin, Hôpital Cochin, 75014 Paris, France; 3 Laboratoire BIO-EC, 91160 Longjumeau, France

**Keywords:** Nrf2, keratinocyte, Langerhans cells, skin, inflammation, photobiomodulation, light, immunomodulation

## Abstract

Photobiomodulation (PBM) is rapidly gaining traction as a valuable tool in dermatology for treating many inflammatory skin conditions using low levels of visible light or near-infrared radiation. However, the physiological regulatory pathways responsible for the anti-inflammatory effect of PBM have not been well defined. Since previous studies showed that nuclear factor-erythroid 2 like 2 (Nrf2) is a master regulator of the skin inflammatory response, we have addressed its role in controlling inflammation by PBM. Primary human keratinocytes (KCs) stimulated with 2,4-dinitrochlorobenzene (DNCB) to mimic pro-inflammatory stress were illuminated with two wavelengths: 660 nm or 520 nm. Both lights significantly reduced the mRNA expression of the DNCB-triggered *TNF-α*, *IL-6*, and *IL-8* cytokines in KCs, while they enhanced Nrf2 pathway activation. PBM-induced Nrf2 is a key regulator of the inflammatory response in KCs since its absence abolished the regulatory effect of light on cytokines production. Further investigations of the mechanisms contributing to the immunoregulatory effect of PBM in inflamed human skin explants showed that 660 nm light prevented Langerhans cells migration into the dermis, preserving their dendricity, and decreased pro-inflammatory cytokine production compared to the DNCB-treated group. This study is the first to report that the PBM-mediated anti-inflammatory response in KCs is Nrf2-dependent and further support the role of PBM in skin immunomodulation. Therefore, PBM should be considered a promising alternative or complementary therapeutic approach for treating skin-related inflammatory diseases.

## 1. Introduction

The skin is a dynamic organ that fulfills multiple functions to maintain the internal environment’s homeostasis. As a primary interface between our body and the outside world, the skin acts as the first line of defense against environmental aggressions (chemicals, microorganisms, and ultraviolet radiation (UV)) [1]. These protections are ensured by the epidermis, the outermost layer of the skin, mainly composed of keratinocytes (KCs), some melanocytes, and immune cells such as resident memory T cells and Langerhans cells (LCs). Long considered passive components of the cutaneous physical barrier, KCs are now described as sentinel cells actively contributing to the immunological barrier of the skin [2,3,4]. KCs play a central role in innate immunity as sensors of danger signals or stress factors through different types of Pattern Recognition Receptors (PRRs), such as Toll-like receptors (TLRs) [2]. The recognition of the cognitive ligands by PRRs on KCs triggers downstream signaling cascades, such as the nuclear factor kappa B (NF-κB) pathway leading to the production of pro-inflammatory mediators such as Tumor necrosis factor-alpha (TNF-α), Interleukin (IL)-1, IL-6, and IL-8. These cytokines and chemokines orchestrate the early immune response of KCs to pathogens and chemical challenges by activating residing immune cells in the skin and recruiting leukocytes to cutaneous sites of inflammation KCs. Therefore, KCs are immunocompetent effector cells that can initiate adequate immune responses to maintain skin homeostasis and defenses and thus have a crucial role in regulating skin inflammation [5].

In physiological conditions, skin cells produce in a controlled manner different reactive species, including reactive species of oxygen (ROS) and nitrogen (RNS) [6,7]. Upon exposure to UV, environmental pollutants, or chemicals, reactive species are produced excessively. The sustained overproduction of these oxidants exceeds the skin’s antioxidant capacities, leading to an imbalance state called “oxidative stress” [8]. Oxidative stress and inflammation are interdependent pathophysiological processes, one of which can be easily induced by the other, sustaining a vicious loop of amplification of both events [9]. To ensure redox homeostasis and prevent the damage from redox active species, the skin has an extensive network of antioxidant defense systems, mainly orchestrated by the nuclear factor erythroid-2-related factor 2 (Nrf2) pathway [6]. It is well established that Nrf2 regulates the gene expression of a broad spectrum of antioxidant enzymes, such as superoxide dismutase (SOD) and glutamate-cysteine ligase catalytic subunit (GCLC), cytoprotective enzymes such as heme oxygenase 1 (HO-1), as well as phase II drug detoxification enzymes such as NAD(P)H quinone oxidoreductase 1 (NQO1) [10,11]. Exposure to oxidative or electrophilic stress leads to the dissociation of Nrf2 from its cytoplasmic inhibitor Kelch-like ECH-associated protein 1 (Keap 1). Nrf2 then translocates into the nucleus and binds to the antioxidant-responsive elements (ARE) of target gene promoters, initiating the transcription of a battery of antioxidants and cytoprotective genes, as cited before [11,12]. In addition to the primary function of Nrf2 as a master regulator of xenobiotic detoxification and redox homeostasis within the cells, recent investigations revealed that Nrf2 also plays an essential role in controlling the inflammatory response [13,14,15]; in particular, skin inflammation [16,17]. As shown in contact hypersensitivity models induced by chemical sensitizers, such as 2,4-dinitrochlorobenzene (DNCB), ear inflammation and lymphocyte proliferation were exacerbated in mice *nrf2*^−/−^ compared to *nrf2*^+/+^, demonstrating an essential role for Nrf2 in controlling allergic contact dermatitis [18]. Another study reveals that Nrf2 controls pro-inflammatory cytokine production in human KCs upon cinnamaldehyde exposure, a well-known skin sensitizer [19]. Nrf2 can control inflammation directly and indirectly [20,21]. Indirect mechanisms to counteract inflammation involve ROS and RNS modulation by Nrf2 and crosstalk of the Nrf2 pathway with redox-sensitive key inflammatory pathways, including NF-κB [21]. Recently, it has been described that Nrf2 can also directly inhibit the transcription of pro-inflammatory cytokine genes such as *IL-6* and *IL-1β* through direct DNA binding close to these genes, independently from ARE, contrary to the widely accepted view that Nrf2 inhibits inflammation through redox control [22].

To control skin inflammation, some safe and effective technologies have recently gained attention in medicine, namely photobiomodulation (PBM). PBM, previously known as Low-Level Light (Laser) Therapy (LLLT), consists of exposing tissues to low-energy visible light or near-infrared radiation (NIR) in order to accelerate tissue repair, reduce inflammation, or alleviate pain. Emerging since the early 2000s, PBM is now a valuable non-invasive tool in cosmetic and medical dermatology for treating numerous skin conditions such as rhytides, scars, ulcers, facial herpes simplex with red light and NIR, rosacea and pigmentary disorders with green light, psoriasis and acne with blue light [23,24]. In vitro studies have reported stimulating effects of visible light on various biological processes in the skin, including the proliferation and differentiation of KCs [25], their migration in wounds [26], and the synthesis of proteins such as filaggrin [27]. Several studies have described specific molecular mechanisms inducing the beneficial effects of light on biological systems. T. Karu’s work showed that cytochrome C oxidase, located in the mitochondrial respiratory chain, is activated in response to red light and NIR [28]. Its absorption of photons leads to the upregulation of cell repair and survival pathways [28]. In vivo rat burn healing models and in vitro inflammation models induced by lipopolysaccharide (LPS) show decreased pro-inflammatory cytokines after exposure to red light and NIR [29,30,31]. Thus far, the endogenous cellular pathways responsible for the well-documented anti-inflammatory effect of PBM have not been fully elucidated. The anti-inflammatory effect of PBM is presumed to be a consequence of ROS modulation. However, it is thought that it is not the only explanation behind this therapeutic property since other signaling pathways are also likely to be involved in reducing inflammation through PBM. Specifically, PBM has been shown to modulate the activity of the NF-κB and mitogen-activated protein kinase (MAPK) pathways, both involved in the regulation of cytokine production and other inflammatory processes [29,32,33]. To date, only a handful of studies have evaluated the effects of PBM on Nrf2 expression and activity. Yadav et al. recently demonstrated activation of the Nrf2 antioxidant pathway associated with an acceleration of burn healing in mice treated at 904 nm [34]. However, there is no strong evidence for a direct link between the therapeutic action of PBM and the Nrf2 signaling during the inflammatory response.

This study aims to understand the cellular mechanisms by which PBM, mainly red light, regulates skin inflammation. We first investigated the role of Nrf2 in the PBM anti-inflammatory response using a two-dimensional model of primary human KCs challenged with DNCB, a contact sensitizer used to mimic pro-inflammatory stress. Herein, we showed that Nrf2 is a crucial factor controlling DNCB-induced pro-inflammatory cytokines production in KCs upon light exposure. Assessment of the PBM effects on LCs activation in human skin explants challenged with the same sensitizer demonstrated the immunoregulatory role of PBM in the skin as it decreases LCs migration into the dermis.

## 2. Materials and Methods

### 2.1. Cell Culture

Human primary epidermal KCs (HPEK) isolated from the juvenile foreskin of multiple donors (>three individuals) were purchased cryopreserved at an early passage from CELLnTEC (Bern, Switzerland). Cells were cultured according to the manufacturer’s protocol in CnT-Prime Epithelial Proliferation medium (CnT-PR) fully supplemented, phenol red-free medium with low calcium formulation (0.07 mM) and a combination of Growth factors and Progenitor Cell Targeted factors maximizing retention of proliferative progenitor cells and minimizing cell differentiation (CELLnTec, Bern, Switzerland). HPEK were seeded after thawing or passaging at a density of 4000 cells/cm^2^ and were incubated at 37 °C in a humidified atmosphere under 5% CO_2_. The medium was replaced every two days. Cells were passaged at ~80% confluency (day 6) after trypsinization with 0.05% trypsin–EDTA solution (Gibco, Paisley, UK). Cells between the second and fourth passages were used for experiments.

### 2.2. Chemical Treatment

When reaching 80% confluency, HPEK were detached using 40 µL/cm^2^ of trypsin-EDTA (0.05%) solution (Gibco, Paisley, UK) and re-plated for at least 18 h in a 24-well plate at a seeding density of 2 × 10^5^ cells per well in 500 µL CnT-PR medium. The next day, the cells were treated with 25 µM DNCB (CAS number: 97-00-7; Sigma-Aldrich, St. Louis, MO, USA) for 3 or 6 h. DNCB was diluted in dimethyl sulfoxide (DMSO, CAS number: 67-68-5, Sigma-Aldrich). The final concentration of the solvent in culture media did not exceed 0.1%. Cytotoxicity was evaluated by measuring the fluorescence of propidium iodide uptake using flow cytometry (Attune^®^ NxT Acoustic Focusing cytometer; Thermo Fisher, Waltham, MA, USA) and analyzed with FlowJo software (version 8.0.2, BD Biosciences, San Jose, CA, USA).

### 2.3. Light Source and Photobiomodulation Treatment

HPEK plated at 2 × 10^5^ cells per well in a 24-well transparent plate were illuminated, as described by Guermonprez et al. [35]. Briefly, the light source was pulsed-wave light-emitting diodes (LEDs) placed in the upper lid of a custom-made device allowing homogeneous and simultaneous irradiation of 12 wells of the plate. LEDs were calibrated with a Qmini 2 spectrometer (RGB Photonics GmbH, Kelheim, Germany) to provide identical light irradiance in each well. Light homogeneity was also controlled for each well (SP90422 Beam Analyser, Ophir Spiricon Europe GmbH, Darmstadt, Germany). Irradiance and homogeneity were measured at the bottom of the wells.

Cells were illuminated at room temperature in air, with two visible pulsed wavelengths separately: 660 nm (red light) or 520 nm (green light), 20 min after DNCB treatment. Light parameters were identical for each wavelength. Cells were illuminated at an irradiance of 12 ± 4 mW/cm^2^ for a total exposure time of 250 s, which conferred a fluence (energy density) of 3 J/cm^2^. The pulse width was fixed at 10 milliseconds, with a duty cycle (DC) of 50%. The distance from the LED to the bottom of the well was 34.5 mm. Cells with no light treatment were used as a negative control (sham group), covered with a black wrap to avoid any light exposition. Following irradiation, cultures were incubated at 37 °C, 5% CO_2_ until required for further analysis.

### 2.4. Gene Silencing

Stealth RNAi™ small interfering RNA (siRNA) technology from Invitrogen (Paisley, UK) was used for *Nrf2* knockdown (NFE2L2 HSS107130), targeting the eight different variants of the transcript. The Forward Transfection Protocol using Lipofectamine^®^ RNAiMax Transfection Reagent (Invitrogen, Carlsbad, CA, USA) was performed on KCs according to the manufacturer’s instructions. Briefly, HPEK were seeded in 24-well plates at a density of 2.5 × 10^4^ per well in 500 µL of CnT-PR medium one day prior to transfection so that they would be 30–50% confluent at the time of transfection. The next day, the transfection mix (siRNA duplex-Lipofectamine^®^ RNAiMAX complexes) was prepared, and cells were transfected with 50 nM of siRNA *Nrf2* (si-Nrf2 group). Mock transfection (no siRNA) and the scrambled non-silencing siRNA (si-random) with no known human gene homology (Stealth™ RNAi Negative Control-Medium GC duplex; Invitrogen, Carlsbad, CA, USA) were performed in each experiment as negative controls. At 48 h after transfection, the transfected HPEK were used for further experiments. The repression of Nrf2 and the expression of Nrf2 target genes were evaluated using Western blot and RT-qPCR, respectively.

### 2.5. RNA Isolation and RT-qPCR

Total RNA was extracted from HPEK using a silica column-based purification with PureLink™ RNA Mini Kit according to the manufacturer’s instructions (Ambion^®^; Invitrogen, Carlsbad, CA, USA). Total RNA yield was quantified by spectrophotometry (Eppendorf BioPhotometer; Hamburg, Germany), and the ratio of absorbance at 260 nm and 280 nm was used to assess the purity of RNA.

A total of 500 ng of RNA was reverse transcribed into cDNA in a total volume of 20 µL with 2.5 µM of oligo (DT) (Promega^®^, Madison, WI, USA), 0.5 mM of dNTP (MP Biomedicals^®^, Santa Ana, CA, USA), 1 U/µL of SuperScript™ IV Reverse Transcriptase (Invitrogen^®^, Waltham, MA, USA), 1 U/µL of RNasin (Promega^®^), 5 mM of Dithiothreitol (DTT; Invitrogen^®^) and 1X Reverse Transcriptase buffer (Invitrogen^®^). Reverse transcription products were diluted at 1:20 in RNase-free water and used for quantitative real-time PCR. Quantitative PCR was carried out with SsoAdvanced™ Universal SYBR^®^ Green Supermix (Bio-Rad, Hercules, CA, USA) using the thermal cycler CFX384™ Touch Real-Time System (Bio-Rad, Hercules, CA, USA). All samples were assayed in duplicate. Primer sequences used for cDNA amplification are listed in Supplementary Table S1. Quantification was performed with CFX Maestro Software (version 2.3, Bio-Rad). The results were normalized to the two house-keeping genes expressions: *gapdh* and *hprt,* and were expressed as the fold increase (i.e., ratio of (1 + E)^−ΔCt^ of target genes/(1 + E)^−ΔCt^ of reference genes, where E is the efficiency and ΔCt is the difference of Ct between treated cells and untreated control cells).

### 2.6. Protein Extraction and Western Blotting

After pretreatment with DNCB and illumination, HPEK were washed twice in phosphate-buffered saline, trypsinized, and centrifuged into a dry pellet. Cells were resuspended in lysis buffer (20 mM Tris HCl (pH 7.4); 137 mM NaCl; 2 mM EDTA; 1% Triton X-100; 2 mM sodium pyrophosphate; 10% glycerol and H_2_O) supplemented with phosphatases and proteases inhibitor cocktail (10 µg/mL aprotinin; 10 µg/mL leupeptin; 1 mM phenylmethylsulfonylfluoride; 1 mM sodium orthovanadate, 100 µg/mL pepstatin and 25 mM β-glycerophosphate). The cells were incubated with the lysis buffer for 20 min in ice and then centrifuged at 15,000 rpm for 20 min at 4 °C. The supernatants containing total cell proteins were stored at −80 °C until use.

After quantification with a BCA protein assay kit (Pierce™, Rockford, IL, USA) for equal protein loading, 40 µg of denatured protein were loaded into 12% SDS-PAGE 1.5 mm gels (TGX-Stain-Free FastCast Acrylamide kit; Bio-Rad, Hercules, CA, USA), electrophoresed and transferred onto polyvinylidene difluoride (PVDF) membrane (Bio-Rad, Hercules, CA, USA). The membrane was blocked for 1 h at room temperature in Tris-buffered saline with 5% nonfat dry milk and incubated overnight at 4 °C with primary rabbit anti-Nrf2 Ab (1:1000 dilution in Tris-buffered saline, 3% BSA; Proteintech^®^, Ref. 16396-1-AP). The next day, the membrane is washed and incubated for 1 h at room temperature with a secondary antibody conjugated to Horseradish peroxidase (HRP) (Cell Signaling Technology, Ref. 7074). Immunoreactive bands were detected by chemiluminescence when adding the ECL substrate (luminol + peroxide solution) (Clarity Max™; Bio-Rad, Segrate, Italy) and visualized using the ChemiDoc XRS + System (Bio-Rad, Hercules, CA, USA). Band densities were quantified with ImageLab software (version 6.1) and normalized to the total protein loaded.

### 2.7. Measurement of Nrf2 Transcription Factor Activity (DNA Binding)

KCs were lysed using the Nuclear Extraction kit (Abcam, ab113474, Cambridge, UK). Quantifying proteins in nuclear extracts was performed with a BCA protein assay kit (Pierce™, Rockford, IL, USA). To quantify Nrf2 activation in nuclear extracts, DNA binding was analyzed using a specific colorimetric ELISA-based Nrf2 Transcription Factor Assay Kit from Abcam (Cambridge, UK) (ab207223), as previously described [36]. Briefly, 27 µg of protein nuclear extracts were incubated for 1 h in a 96-well plate where a specific double-stranded DNA sequence containing the Nrf2 consensus binding site (5′-GTCACAGTGACTCAGCAGAATCTG-3′) was immobilized. Active Nrf2 present in the nuclear extract specifically binds to the oligonucleotide. After washing, the plate was incubated for 1 h with the appropriate primary Ab, washed, and then incubated with horseradish peroxidase-conjugated secondary Ab (1:1000) for 1 h at room temperature. The colorimetric readout was quantified by spectrophotometry at 450 nm.

### 2.8. Cytokines Dosage

KCs were stimulated with 25 µM of DNCB with or without illumination and incubated for 6 h at 37 °C in a humidified atmosphere under 5% CO_2_. The level of produced pro-inflammatory mediators (IL-1α, IL-1β, IL-6, IL-8, TNF-α, IP-10, MIP-3α, and MCP-1) was measured in the supernatants, as previously described [37] by Meso Scale Discovery^®^ (Meso Scale Diagnostics, Rockville, MD, USA) multiplex assay, according to manufacturer’s instructions. Briefly, the 96-well U-PLEX plate was coated with the different linker-coupled capture antibodies where the linkers self-assemble onto unique spots in each well of the plate. After analytes in the sample bound to the capture reagents, detection antibodies conjugated with electrochemiluminescent labels (MSD GOLD™ SULFO-TAG, Meso Scale Diagnostics, Rockville, MD, USA) bind to the analytes to complete the sandwich immunoassay. The plate was then placed into an MSD instrument (Meso QuickPlex SQ 120MM, Meso Scale Diagnostics, Rockville, MD, USA) where the level of cytokines in the sample was measured and calculated with Discovery Workbench^®^ analysis software (version 4, MSD, Rockville, MD, USA) compared to the standard calibrator.

### 2.9. Human Skin Explants Preparation and Treatment

Skin biopsies were processed by BIO-EC Laboratory (Longjumeau, France). BIO-EC Laboratory has authorization from the Bioethics group of the general director services of the French research and innovation ministry (registered under n°DC 2022-5373) to use human skin from surgical waste since 5 May 2010. The study was performed following the Declaration of Helsinki after the patients had given consent to use their skin samples by BIO-EC Laboratory.

Full-thickness human skin specimens were obtained from abdominal plastic surgery of a 42 years-old healthy Caucasian woman with a skin phototype II-III. Human skin explants were prepared, as previously described [35]. Briefly, the subcutaneous fat was removed from the skin, and explants of around 12 ± 1 mm in diameter were cut out using a circular scalpel; each condition was tested in triplicate (*n* = 3 explants per batch). Skin explants were then cultured on nitrocellulose filters placed on stainless steel grids at the air–liquid interphase, epidermal side up. The grids were then placed in 6-well culture plates containing proprietary explant medium developed and owned by the BIO-EC. Explants were cultured at 37 °C in 5% CO_2_ humidified air. After 24 h of stabilization, 6 µL of DNCB at 0.25% (*w*/*v*) diluted in 20% DMSO was applied epicutaneously onto the skin explants (6 µL/explant) in pre-evaluated nontoxic concentration. Red light (660 nm) treatment was then dispensed in a pulsed mode (pulse period = 10 milliseconds, DC = 50%) for 250 s using Lightinderm lighting device (Lightinderm, Paris, France). The lighting devices were held as close as possible to the skin explant without any contact with the skin surface. The control explants did not receive any DNCB or lighting treatment. Explants were harvested 24 h after DNCB and light exposure. Every explant was cut in two parts: half were frozen at −80 °C for immunostaining, another half was fixed in buffered formalin for evaluation of the skin morphology according to Masson’s trichrome protocol, Goldner variant [38]. The microscopical observations were realized using a Leica DMLB or a BX43 Olympus microscope. Pictures were digitized with a numeric DP72 or DP74 Olympus camera (Olympus, Tokyo, Japan) with cellSens data storing software (Olympus, Tokyo, Japan).

### 2.10. Immunostaining of Skin Explants

The frozen samples were cut into 7 μm thick sections using a Leica CM 3050 cryostat. Sections were then mounted on Superfrost^®^ (London, UK) plus salinized glass slides. Langerin (CD207) immunostaining was performed on frozen skin sections with a monoclonal anti-langerin antibody (Beckman Coulter, ref. PN IM3449, clone DCGM4, Brea, CA, USA), diluted at 1:200 in PBS-BSA 0.3%-Tween 20 at 0.05% and incubated for 1 h at room temperature. The staining was revealed by goat anti-mouse IgG, cross-adsorbed secondary antibody, Alexa Fluor™ 488 (Invitrogen, ref. A11001, Waltham, MA, USA). The nuclei were counterstained using propidium iodide. The immunostaining was assessed by microscopical observation. The absolute number of langerin-positive cells in the epidermis was counted in 2 different sections from the 3 explants of each batch and was related to the length of the epidermis to obtain the number of langerin-positive cells/cm of epidermis.

TNF-α immunostaining was performed on formalin-fixed paraffin-embedded skin sections with a polyclonal anti-TNFalpha antibody (Novus biologicals, ref. NBP1-19532, Englewood, CO, USA) diluted at 1:100 in PBS-BSA 0.3%-Tween 20 (0.05%), for 1 h at room temperature, using Vectastain Kit Vector amplifier system avidin/biotin, and revealed by VIP (Vector laboratories, Ref. SK-4600, Burlingame, CA, USA), a substrate of peroxidase giving violet staining once oxidized. Quantifying TNF-α expression in the epidermis was assessed by image analysis and calculation of the surface percentage of the epidermis covered by the staining (stained surface percentage) using cellSens software (version 3.2, Olympus, Tokyo, Japan).

### 2.11. Statistical Analysis

Statistical Analyses were performed using GraphPad Prism^®^ software (version 8,GraphPad Software-Dotmatics, San Diego, CA, USA). Repeated-measures two-way ANOVA followed by Tukey’s multiple comparison post hoc test was used to compare different groups, and the homoscedasticity of residuals was checked. Data are expressed as means ± SEM and considered statistically different when the *p*-value < 0.05.

## 3. Results

### 3.1. PBM Regulates Pro-Inflammatory Mediators in Stressed KCs

**Figure 1:**
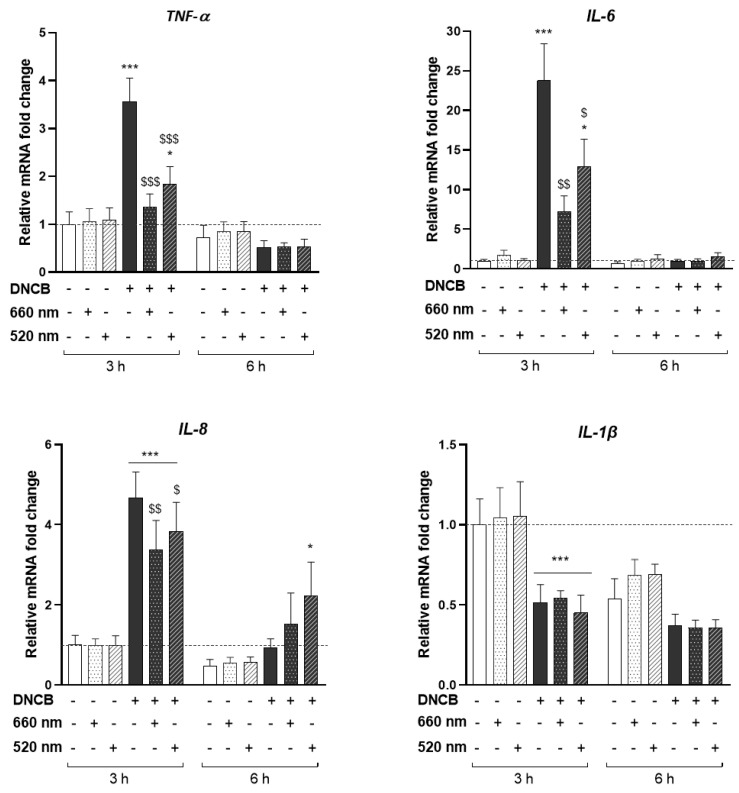
PBM regulates pro-inflammatory DNCB-induced cytokines in KCs. KCs were treated with DNCB or vehicle, with or without red (660 nm) or green (520 nm) light illumination (3 J/cm^2^, 250 s). Pro-inflammatory cytokines gene expression: *TNF-α*, *IL-6*, *IL-8*, and *IL-1β*, were measured by RT-qPCR 3 and 6 h after DNCB and light treatment. Results are expressed as fold change. Data represent mean ± SEM of 5 independent experiments. Two-Way ANOVA followed by Tukey post hoc test, * *p* < 0.05; *** *p* < 0.001 vs. the relative control at 3 h or 6 h; $ *p* < 0.05; $$ *p* < 0.01; $$$ *p* < 0.001 vs. DNCB at 3 h.

HPEK were stimulated with 25 µM of DNCB, an electrophilic chemical sensitizer used to induce the pro-inflammatory cellular stress. The concentration of DNCB leading to 70% of cell viability was selected. KCs were then illuminated with two different wavelengths separately: 660 nm (red light) or 520 nm (green light), at 3 J/cm^2^ for a total exposure time of 250 s. First, we investigated the ability of these two wavelengths to control the inflammatory response of KCs at the pre-selected dosimetry. The mRNA level of different pro-inflammatory cytokines was assessed by RT-qPCR after 3 or 6 h of DNCB stimulation and light exposure (Figure 1).

LED exposure did not modify the expression of any studied cytokine compared to untreated cells, at basal state (Figure 1), while DNCB induced a significant increase in the expression of *TNF-α* (3.56-fold increase), *IL-6* (23.76-fold increase), and *IL-8* (4.68-fold increase) 3 h post-stimulation. In contrast, *IL-1β* expression was downregulated by 52% after 3 h of DNCB treatment (Figure 1). Interestingly, red light significantly suppressed the expression of the DNCB-triggered *TNF-α*, *IL-6*, and *IL-8* mRNAs by 62%, 69%, and 28%, respectively, 3 h post-illumination. Green LED exposure likewise significantly decreased the mRNA expression of *TNF-α* by 49%, *IL-6* by 46%, and *IL-8* by 18% compared with DNCB-stimulated conditions (Figure 1). No significant modulation of *IL-1β* expression due to the illumination was observed. *TNF-α*, *IL-6*, and *IL-8* mRNAs expression were not modified by light upon 6 h of DNCB stimulation. Furthermore, only *IL-8* expression following DNCB plus green light exposure was different from the control evaluated at 6 h post-stimulation (Figure 1).

Altogether, these results showed that PBM did not modulate the transcription of pro-inflammatory cytokines in KCs at the basal state. However, red and green LEDs decreased the expression of cytokines induced by DNCB.

### 3.2. PBM Enhances Nrf2 Pathway Activation in DNCB-Stimulated KCs

**Figure 2:**
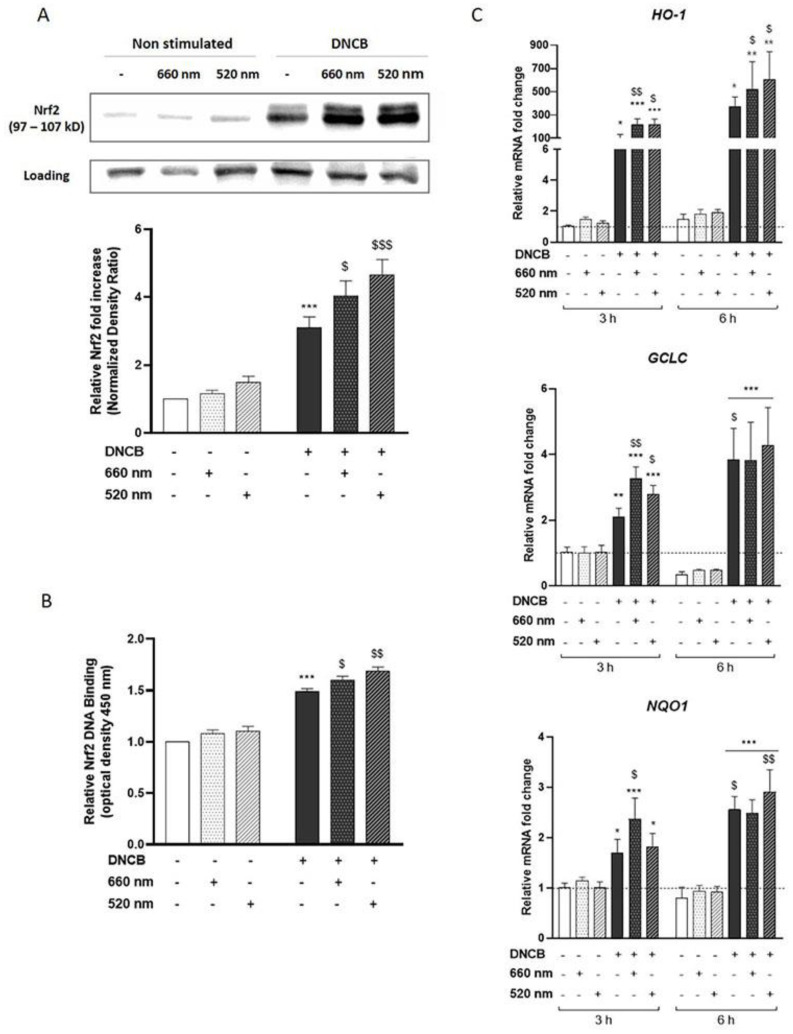
PBM enhances Nrf2-pathway activation in DNCB-stimulated KCs. KCs were treated with vehicle (DMSO 0.1%) or DNCB (25 µM) and illuminated with either wavelength: 660 nm (red light) or 520 nm (green light). (**A**) Nrf2 accumulation was assessed by Western blot 3 h after illumination. Histograms correspond to densitometric analysis relative to controls and are adjusted to the stain-free blot. (**B**) ELISA-based measurement of DNA-binding activity for activated Nrf2 in the nuclear extract, 4 h after KCs treatment. (**C**) Nrf2 target antioxidant gene expression: *HO-1*, *GCLC*, and *NQO1*, were assessed by RT-qPCR, 3 h or 6 h after light treatment. Data represent mean ± SEM of at least five independent experiments. Two-Way ANOVA followed by Tukey post hoc test, * *p* < 0.05; ** *p* < 0.01; *** *p* < 0.001 vs. the relative untreated control at 3 h or 6 h; $ *p* < 0.05; $$ *p* < 0.01; $$$ *p* < 0.001 vs. DNCB at 3 h.

As Nrf2 has been described as a key factor in regulating skin inflammation [17,18,19,39], we investigated the activation of the Nrf2 pathway in response to light exposure. First, Nrf2 protein accumulation was assessed by Western blot after 3 h of DNCB and light treatment. Our results showed that DNCB significantly increased Nrf2 accumulation compared to untreated control KCs. Red and green LEDs indifferently enhanced Nrf2 accumulation by 30% and 50%, respectively, compared to DNCB-treated KCs (Figure 2A). These observations were associated with a slight increase in Nrf2 DNA-binding to an ARE consensus sequence upon exposure to red (7.3%) and green (12.7%) light compared to DNCB-treated KCs (Figure 2B), showing an increase in the transcriptional activity of Nrf2. Since Nrf2 was shown to be active in the nucleus, the next step was to assess the expression of several Nrf2-target genes by RT-qPCR. DNCB increased mRNA expression of *HO-1* (103-fold increase), *GCLC* (2.09-fold increase), and *NQO1* (1.69-fold increase), while PBM further enhanced the expression of these Nrf2-target genes after illumination with red (113.6%, 53.9%, and 40%, respectively) or green (*HO-1* by 108.3% and *GCLC* by 31.3%) light at 3 h of KCs stimulation (Figure 2C). However, the light-induced upregulation of Nrf2-target genes did not persist 6 h after cell illumination (Figure 2C).

Taken together, these data revealed that PBM reinforced the DNCB-induced accumulation and activation of Nrf2 and slightly increased the expression of the antioxidant target genes.

### 3.3. Nrf2 Mediates the Anti-Inflammatory Response of the Red Light in KCs

**Figure 3:**
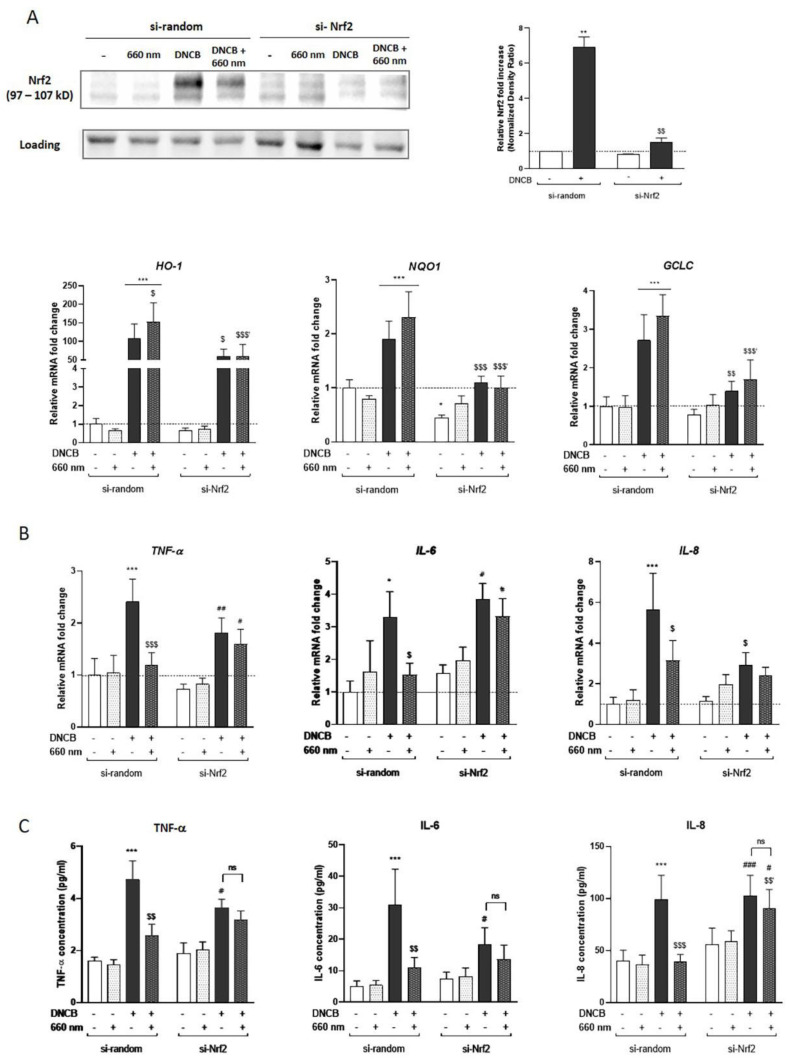
The anti-inflammatory effect of red light in KCs is Nrf2-dependent. KCs were transfected for 48 h by si-Nrf2 or si-random, and then treated with DNCB and exposed to red light. (**A**) Nrf2 accumulation in the knockdown model was assessed by Western blot 3 h after treatment. The blot is representative of 3 independent experiments. Histograms correspond to densitometric analysis relative to untreated si-random and are adjusted to the stain-free blot. Quantifying the expression of Nrf2 target genes encoding *HO-1*, *NQO1*, and *GCLC* in transfected KCs were assessed using RT-qPCR 3 h after treatment. Induction values were the ratio between gene expressions in treated cells versus gene expression in untreated si-random control. (**B**) Pro-inflammatory cytokines gene expression: *TNF-α, IL-6* and *IL-8*, were assessed by RT-qPCR, 3 h after light treatment. Results are expressed as fold change compared to the untreated si-random control. (**C**) The inflammatory cytokines level was determined by Meso Scaled Discovery technology in the supernatant of KCs after 6 h of exposure to DNCB with or without red light illumination. Reported data are mean ± SEM of 6 independent experiments. Two-Way ANOVA followed by Tukey post hoc test, * *p* < 0.05; ** *p* < 0.01; *** *p* < 0.001 vs. untreated si-random; $ *p* < 0.05; $$ *p* < 0.01; $$$ *p* < 0.001 vs. DNCB-treated si-random, $$′ *p* < 0.01; $$$′ *p* < 0.001 vs. DNCB + 660 nm-treated si-random; # *p* < 0.05, ## *p* < 0.01, ### *p* < 0.001 vs. untreated si-Nrf2. ns means non-significant different.

Based on the literature showing the anti-inflammatory role of Nrf2 in KCs [19] and the observed effect of PBM controlling pro-inflammatory cytokines (Figure 1), we suggested that Nrf2 activation might be involved in the control of the PBM-mediated inflammatory response upon DNCB exposure. To address this hypothesis, Nrf2 was invalidated in KCs by siRNA transfection strategy (si-Nrf2) and focused specifically on the red light response after DNCB treatment. Nrf2 accumulation in DNCB-treated Nrf2-invalidated KCs was abrogated by 78.3% compared to its relative si-random control (Figure 3A). In a similar manner, the mRNA expressions of *HO-1*, *NQO1*, and *GCLC* were significantly downregulated by 60.7%, 53.2%, and 49.4%, respectively, in the DNCB-stimulated and light-exposed group (Figure 3A).

To study the response of KCs to red light exposure in the absence of Nrf2, the mRNA expression and secretion of three pro-inflammatory mediators were assessed. As previously described in Figure 1, the red light attenuated DNCB-induced *TNF-α*, *IL-6*, and *IL-8* expression in the si-random control group (Figure 3B). Interestingly, in the absence of Nrf2, illumination of KCs with red light did not reduce the expression of these cytokines in response to DNCB (Figure 3B). Furthermore, these results correlated with the level of cytokines measured in the supernatant by electroluminescence (Figure 3C). While red light in the control group significantly inhibited DNCB-induced TNF-α, IL-6, IL-8 and also IL-1α, and IP-10 production, red LED failed to suppress the release of these pro-inflammatory mediators in si-Nrf2 KCs (Figure 3C and Figure S2). These findings implied that the observed anti-inflammatory effect of red light upon DNCB stimulation depends, at least in part, on Nrf2. The production of other mediators such as IL-1β, MIP-3α, and MCP-1 was tested, but their secretion level at 6 h post-stimulation was below the assay’s detection limit (data not shown). In our study, Nrf2 downregulation in KCs increased the basal level of *IL-1β* transcription at the steady state (Figure S1). IL-1α was the only cytokine whose secretion was significantly increased in the absence of Nrf2 in the untreated group or in response to DNCB (Figure S2), suggesting that Nrf2 might play a role in the regulation of IL-1β expression as well as IL-1α production in KCs.

Overall, our results showed that PBM response controlling DNCB-induced pro-inflammatory cytokines and chemokines is, at least partially, dependent on Nrf2.

### 3.4. PBM Reduces DNCB-Induced LCs Activation and TNF-α Production in the Epidermis

To investigate the mechanisms of the immunomodulatory response induced by PBM in a more physiologically relevant model, we used an ex vivo model of human skin explants treated with the DNCB for 24 h and exposed to red light. As shown in the microscopical histological evaluation, the solvent (DMSO 20%), the red-light exposure (660 nm, 12 mW/cm^2^ for 250 s), and the DNCB topical application (0.25%) did not induce any significant modification or morphological alterations in the epidermal or dermal structures (Figure S3).

**Figure 4:**
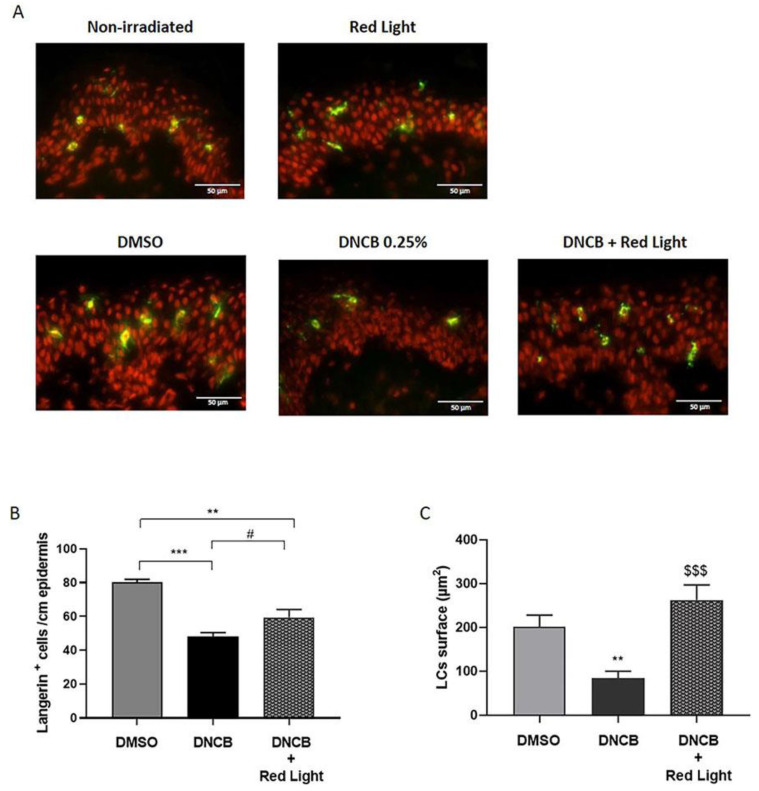
Red light decreases DNCB-induced LCs activation in the epidermis. Human skin explants were topically treated with the excipient (DMSO 20%) or DNCB (0.25% *w*/*v*) with or without red light exposure (660 nm, 3 J/cm^2^, 250 s). (**A**) Langerin immunostaining was performed on frozen skin sections 24 h after DNCB treatment and revealed by AlexaFluor 488 (green). The nuclei were counterstained using propidium iodide (red). Scale bars represent 50 µm. (**B**) The absolute number of langerin-positive cells in the epidermis was counted at 2 different zones for the 3 explants of each batch. Histogram represents the mean ± SEM of 6 measures of langerin-positive cells/cm epidermis. (**C**) Langerin-positive cells total surface was measured by ImageJ software (version 1.8). Histogram represents the mean ± SEM of cell surface of 10 different measures. ANOVA followed by Tukey post hoc test; ** *p* < 0.01; *** *p* < 0.001 vs. DMSO-treated control group; # *p* = 0.06, $$$ *p* < 0.001 vs. DNCB-treated group.

Upon activation, LCs, professional antigen-presenting cells residing in the epidermis, undergo morphological changes and migrate to skin-draining lymph nodes to initiate an appropriate immune response in response to sensitizers such as DNCB [40,41]. To follow LCs migration from the epidermis into the dermis in response to DNCB and light exposure, we immunostained the langerin (CD207), a well-known marker of LCs. The topical application of DNCB (0.25%) induced a significant decrease in the number of langerin-positive cells (CD207^+^) in the epidermis compared to the DMSO excipient batch (40%; *p* < 0.001) (Figure 4A,B). Furthermore, LCs in the DNCB-treated explants had a more constricted and rounded shape, associated with less frequent dendrites than those from the DMSO-treated control group (Figure 4C and Figure S4). DNCB also increased TNF-α expression in the epidermis by 26% (Figure S5) and augmented the level of IL-6, Il-1α, and IL-1β (*p* < 0.05) secretion by skin explants compared to the DMSO-treated group (Figure S6). The exposure of explants to red light increased by 23% the number of langerin-positive cells in the epidermis compared to the DNCB alone with a *p*-value = 0.06 (Figure 4A,B). The LCs surface of red light-exposed tissues following DNCB treatment was significantly increased (DNCB mean = 84 µm^2^; DNCB + red light mean = 263 µm^2^; *p* < 0.001) with a higher dendricity (Figure 4C and Figure S4). These observations were supported by a significant decrease of 26% in TNF-α expression level in the epidermis of light-exposed explants (Figure S5). Moreover, our results suggest that exposure to red light slightly decreased pro-inflammatory cytokine secretion in skin explants compared to the DNCB-treated group (*p* < 0.1) (Figure S6), although this effect did not reach statistical significance at the conventional level, likely due to the small number of replicates. Additional studies with larger sample sizes and more statistical power are needed to confirm these findings.

Taken together, these results implied that red light might modify the skin environment by reducing pro-inflammatory cytokine production induced by DNCB, and prevented LCs activation by inhibiting their migration and maintaining their dendrites.

## 4. Discussion

Inflammation is a major contributor to many skin diseases, and the ability to reduce inflammation is of paramount importance in medicine. PBM is an increasingly popular therapy for treating skin inflammation and many dermatological diseases using low-energy visible light or NIR [42,43,44]. Although it has recently gained considerable recognition for its potential to reduce inflammation, the mechanisms underlying the anti-inflammatory effect of PBM remain unclear. We conducted this study to understand the cellular pathways by which PBM can regulate skin inflammation.

Since KCs constitute the major cell population of the epidermis and are a source of inflammation involved in many skin pathologies [2,4,45], we chose to work on a two-dimensional model of primary human KCs to study the benefic effect of PBM. To mimic the pro-inflammatory stress, KCs were exposed to DNCB, and then, the modulation of the inflammatory response by red and green lights was addressed. DNCB is an electrophilic chemical classified as an extreme contact sensitizer [46]. It induces a pro-inflammatory microenvironment and subsequently promotes a complete immune response. Thus, it is widely used for challenging rodents’ skin in contact hypersensitivity [18,47] or atopic dermatitis [48,49] models. It has also been used in several in vitro studies characterizing the skin sensitization response to allergens in primary KCs or a well-known KCs cell line, HaCaT [50,51]. As previously described, our data showed an increase in the mRNA expression levels of *TNF-α*, *IL-6*, and *IL-8* in response to DNCB. It is consistent with previous reports showing that DNCB treatment specifically increases IL-6 secretion in HaCaT cells as well as the transcription of *TNF-α*, *IL-6*, and *IL-8* in primary KCs [52,53].

In our study, the illumination with red (660 nm) or green (520 nm) LEDs at 3 J/cm^2^ for 250 s downregulated the expression of the DNCB-induced cytokines, indicating the anti-inflammatory role that plays PBM in KCs. There is growing evidence supporting that PBM serves as a regulator of inflammation. In DNCB-induced atopy mouse model, illumination with visible red (650 nm) alleviates symptoms of atopic dermatitis and restores cytokine levels such as IL-6 and TNF-α to normal [54]. An in vivo study of a rat burn healing model daily irradiated with 660 nm low-power laser (10 J/cm^2^, 20 s) for 7 consecutive days showed inhibition of IL-6 expression at the site of injury, associated with a decrease in oxidative stress and an improved cutaneous antioxidant profile, thus, accelerating the repair of burn wounds [31]. In the same model, green LED (550 nm, 60 J/cm^2^, 10 s), a less documented light than red, was able to reduce inflammatory cells’ infiltration to the burn site, composed predominantly of neutrophils and lymphocytes [55]. In vitro studies using TLR-agonists such as bacterial LPS to induce inflammation further supported the anti-inflammatory effect of PBM. The illumination of human gingival fibroblasts with 635 nm LED suppressed the LPS-induced IL-6, IL-8, and prostaglandin E_2_ release, correlated with modulation of MAPK pathway activation [29]. In the same way, LPS-induced *IL-6* and *IL-8* mRNA expressions were significantly decreased by low-level 805 nm (NIR) laser irradiation of the oral squamous epithelial carcinoma cell line (Ca9-22) [56]. Fernandes et al. showed a reduction of TNF-α production in murine J774 macrophage-like cells activated by LPS and IFNγ and illuminated with a 660 nm laser (15 mW/cm^2^, 7.5 J/cm^2^). However, the authors reported an up-regulation of IL-6 by light, in contrast to our result [57]. Although they used the same red wavelength as in our study, at a power density and fluence ranges close to those we used, the difference in IL-6 expression may be explained by the difference in cell type and species (human KCs vs. murine macrophage-like cells), the difference in total light exposure time (250 s vs. 20 s), and the time point of the mRNA expression measurement (3 h vs. 24 h, where the measured expression is no longer a direct effect of light but rather a secondary effect to other cellular processes). IL-6 and IL-8 are “secondary” pro-inflammatory mediators that act in an autocrine manner. Their function is mediated by “primary” cytokines, such as TNF-α, a key regulator of inflammation secreted in response to stimuli [2]. Pro-inflammatory cytokines and chemokines, including IL-6, IL-8, and TNF-α, play a critical role in the initiation and progression of many chronic inflammatory skin diseases, such as psoriasis or atopic dermatitis [58]. Thus, control of their production by PBM can regulate the amplitude and duration of the inflammatory response and modify the course of the disease.

Different mechanisms have been proposed to explain the anti-inflammatory effects of PBM [59,60,61], but most of these observations lack strong evidence concerning the exact involvement of these mechanisms in the therapeutic action of PBM. The Nrf2 pathway is a key regulator of the response to oxidative stress and inflammation. It plays a pivotal role in the regulation of cellular defense mechanisms, including the promotion of an anti-inflammatory response [14,16,17]. To date, only a few studies have evaluated the effects of PBM on Nrf2 expression and activity. Trotter et al. reported a rapid accumulation and nuclear translocation of Nrf2 after treating the human monocytic cell line, THP-1, with blue light (45 J/cm^2^), followed by induction of *HO-1* expression, an Nrf2-target cytoprotective enzyme. These observations were associated with reduced LPS-induced TNF-α and IL-8 secretion [62]. The same authors also found that blue light treatment induced upregulation of Nrf2 in A431 epidermoid carcinoma cells and significantly increased levels of *HO-1* [63]. Furthermore, Sohn et al. showed an upregulation of *Nrf2* gene expression and protein accumulation upon treatment of RANKL-stimulated mouse bone marrow-derived macrophages with 635 nm LED [64]. Yadav et al. recently demonstrated activation of the Nrf2 pathway associated with an acceleration of burn healing in mice treated at 904 nm [34]. In line with all these observations, we showed an activation of the Nrf2 signaling by red and green lights in DNCB-stimulated KCs. Indeed, 660 nm and 520 nm LEDs further enhanced Nrf2 accumulation and nuclear translocation compared to the DNCB-treated group. They also increased Nrf2-target genes expression, *HO-1, NQO1,* and *GCLC.* To our knowledge, this effect of PBM on Nrf2 pathway activation has not been previously reported in KCs. Since Nrf2 is a key regulator of ROS production, we measured in our study hydrogen peroxide (H_2_O_2_) in stimulated KCs. No significant modifications of H_2_O_2_ level were observed after DNCB treatment with or without the illumination of KCs (data not shown), suggesting that PBM-induced Nrf2 does not seem to regulate inflammation through ROS modulation. Moreover, our study is the first to report that Nrf2 is an essential factor for controlling KCs inflammatory response by the red light, a more commonly used PBM protocol than the green light to treat inflammation in vivo and in vitro. Indeed, in the absence of Nrf2, the illumination of KCs did not reduce the TNF-α, IL-6, or IL-8 production in response to DNCB.

Since the crosstalk between KCs and LCs is essential for programming LCs response and determining the appropriate adaptive immune response (immunogenic or tolerogenic) [40,41,65], we addressed the immunoregulatory role of PBM in a more physiologically relevant model. We assessed the effect of red LED on LCs in human skin explants treated epicutaneously with DNCB to induce inflammation. In our study, DNCB induced more rounded shrunken LCs with fewer dendrites and promoted their migration from the epidermis into the dermis, indicating activation of LCs. These morphological changes were associated with increased pro-inflammatory cytokine secretion in skin explants and augmented epidermal expression of TNF-α, a marker of skin inflammation and known to play a role in stimulating LCs migration. These findings corroborate previous reports demonstrating an LCs mobilization induced by skin sensitization to DNCB, though TNF-α was not an indispensable requirement for LCs migration in mouse skin [53,66,67]. We showed that the exposure of skin explants to red light (660 nm) prevented the DNCB-induced LCs modifications and migration. It also decreased the expression of TNF-α in the epidermis and tended to regulate the level of some pro-inflammatory cytokine secretion. Thus, this result provides evidence for the immunomodulatory effect of PBM that might contribute to its anti-inflammatory activity preventing allergen sensitization in atopic or allergic dermatitis. Visible red (633 nm) LED has been previously shown to suppress the Th2 immune response induced by topical sensitization of mice skin with a protein antigen, ovalbumin. In the same study, PBM reduced the expression of CD24, a costimulatory surface molecule, among others expressed by LCs [68]. A previous report showed that a single exposure of mouse skin to NIR (30 J/cm^2^) reversibly suppressed the number of LCs that became round with shortened dendrites, contrary to our observations. However, the reduced density of LCs was correlated to a failure of the skin to induce a contact hypersensitivity reaction, suggesting that NIR induced local immunosuppression, probably by initiating an antigen-specific Treg response [69]. Another study demonstrates that NIR (90 J/cm^2^) can modulate immune response in vivo by activating LCs [70]. Although these studies support the ability of PBM to modulate LCs response, the difference in the LCs activation and mobilization can be attributed to the difference in the wavelengths and the energy densities, which were relatively high compared to the one we used. By impairing cytokine release in the skin microenvironment, PBM may alter the activation of LCs. As Nrf2 activation can control the skin microenvironment [17,20] by regulating the activation of KCs and LCs, it may modulate their ability to elicit an adaptative immunity [18,71]. It is not excluded that PBM may also alter the expression of surface molecules of LCs by a direct mechanism. Further research is necessary to explore the exact mechanisms driving the effects of PBM on LCs activation and to describe the phenotype of LCs in response to PBM to discuss its potential therapeutic applications.

## 5. Conclusions

In summary, the data presented here support previous studies on the anti-inflammatory effect of PBM. Our findings provide new evidence that the PBM’s beneficial activity in human KCs with an inflammatory response is at least dependent on the Nrf2 pathway. Furthermore, the therapeutic effect of PBM in response to DNCB does not seem to involve regulation of the level of H_2_O_2_ in KCs, but rather a modulation of the skin immune response, probably through the regulation of innate cytokines that are crucial for initiating and perpetuating inflammation. PBM controls TNF-α expression in the epidermis of human explants and prevented the activation of LCs and their migration into the dermis in response to chemical stress.

Altogether, our results support the regulatory function of PBM modulating key immune cells in the epidermis. It acts as an inhibitor of the primary inflammatory response induced by KCs, mainly through the Nrf2 pathway, and reduces LCs activation. Therefore, PBM has a promising future as an effective non-invasive anti-inflammatory therapeutic for skin-related inflammatory diseases, especially those in which Nrf2 has been shown to play a pivotal role as a regulator of inflammation, such as psoriasis, wound healing, or allergic contact dermatitis.

## Data Availability

The data presented in this study is contained within this manuscript or Supplementary Material.

## Supplementary Materials

The following supporting information can be downloaded at: https://www.mdpi.com/article/10.3390/antiox12030766/s1, Table S1: Oligonucleotide primer sequences used for RT-qPCR; Figure S1: *IL-1β* mRNA expression in KCs Knocked down for Nrf2 following DNCB and red LED treatment; Figure S2: IL-1α and IP-10 levels secreted by KCs knocked down for Nrf2 in response to DNCB and red light exposure; Figure S3: Optical microscopy evaluation of skin explants; Figure S4: Representative images of LCs dendricity; Figure S5: Red light decreases DNCB-induced TNF-α expression in the epidermis; Figure S6: Red light slightly decreases DNCB-induced pro-inflammatory cytokines secretion in the skin explants.

## Abbreviations

ARE: Antioxidant responsive element; DMSO: Dimethyl sulfoxide; DNCB: 2,4-dinitrochlorobenzene; GCLC, Glutamate-cysteine ligase catalytic subunit; HPEK, Human primary epidermal keratinocytes; HO-1, Heme oxygenase 1; IL, Interleukin; KCs, Keratinocytes; LCs, Langerhans cells; LED, Light-emitting diode; MAPK, mitogen-activated protein kinase; MSD, Meso scaled discovery technology; NF-κB, Nuclear factor kappa; NIR, Near-infrared radiation; NQO1, NAD(P)H quinone oxidoreductase 1; Nrf2, Nuclear factor erythroid-2-related factor 2; PBM, Photobiomodulation; ROS, Reactive oxygen species; TLR, Toll-like receptor; TNF-α, Tumor necrosis factor-alpha; UV, Ultraviolet radiation.
