# Supplementary material for: Photobiomodulation Controls Keratinocytes Inflammatory Response through Nrf2 and Reduces Langerhans Cells Activation
Source: Antioxidants (Basel). 2023 Mar 21;12(3):766. doi: 10.3390/antiox12030766 (PMC10045240; doi:10.3390/antiox12030766)
Supplement: Supplementary file 1 [file antioxidants-12-00766-s001.zip › antioxidants-2190024-supplementary.pdf]

Supplementary data

Table S1. Oligonucleotide primer sequences used for qRT-PCR.

Gene Symbol	Gene Name	NCBI Gene Accession Number	Forward Primer Sequence	Reverse Primer Sequence
GAPDH	*Glyceraldehyde-3-phosphate dehydrogenase*	NM_001357943	ACCACAGTCCATGCCATCAC	TCCACCACCCTGTTGCTGTA
GCLC	*Glutamate-cysteine ligase catalytic subunit*	NM_001197115	ACATGGAAGTGGATGTGGACACC	CCTCATCCATCTGGCAACTGTCA
HO-1	*Heme oxygenase-1*	NM_002133	GGCCTGGCCTTCTTCACCTT	GAGGGGCTCTGGTCCTTGGT
HPRT	*Hypoxanthine-guanine phosphoribosyltransférase*	NM_000194	GCCAGACTTTGTTGGATTTG	CTCTCATCTTAGGCTTTGTATTTTG
IL-1β	*Interleukin 1 beta*	NM_000576	ACAGACCTTCCAGGAGAATG	GCAGTTCAGTGATCGTACAG
IL-6	*Interleukin 6*	NM_001371096	TCAATGAGGAGACTTGCCTG	GATGAGTTGTCATGTCCTGC
IL-8	*Interleukin 8 / C-X-C motif chemokine ligand 8 (cxcl8)*	NM_001354840	TCTCTTGGCAGCCTTCCTGA	TGGGGTGGAAAGGTTTGGAG
NQO1	*NAD(P)H quinone dehydrogenase 1*	NM_001025434	GGGCAAGTCCATCCCAACTG	GCAAGTCAGGGAAGCCTGGA
NRF2	*Nuclear factor erythroid-2-like 2 (NFE2L2)*	NM_001313903	CTTGGCCTCAGTGATTCTGAAGTG	CCTGAGATGGTGACAAGGGTTGTA
TNF-α	*Tumor necrosis factor*	NM_000594	CCTCTCTCTAATCAGCCCTCTG	GAGGACCTGGGAGTAGATGAG

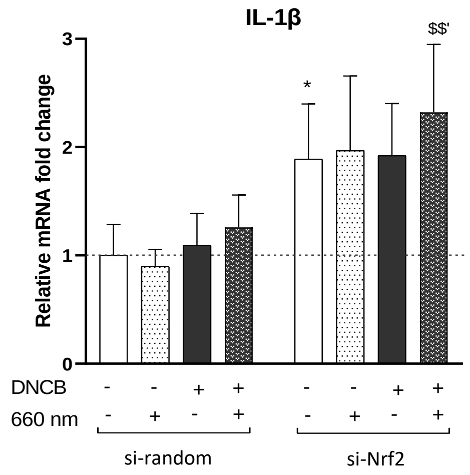

Figure S1.

***IL-1β* mRNA expression in KCs knocked down for Nrf2 following DNCB and red LED treatment.**
KCs were transfected for 48 h by siRNA directed against Nrf2 (si-Nrf2) or random siRNA (si-random). KCs were then treated with DNCB (25μM) and exposed to red light (660 nm, 3 J/cm^2). Pro-inflammatory cytokine *IL-1β* gene expression was assessed by RT-qPCR 3 h after light treatment. Results are expressed as fold change compared to the untreated siRNA control random. Reported data are mean ± SEM of 6 independent experiments. Two-Way ANOVA followed by Tukey post-hoc test, * p < 0.05 vs. untreated si-random; $$' p <0.01 vs. DNCB + 660 nm- treated si-random group.

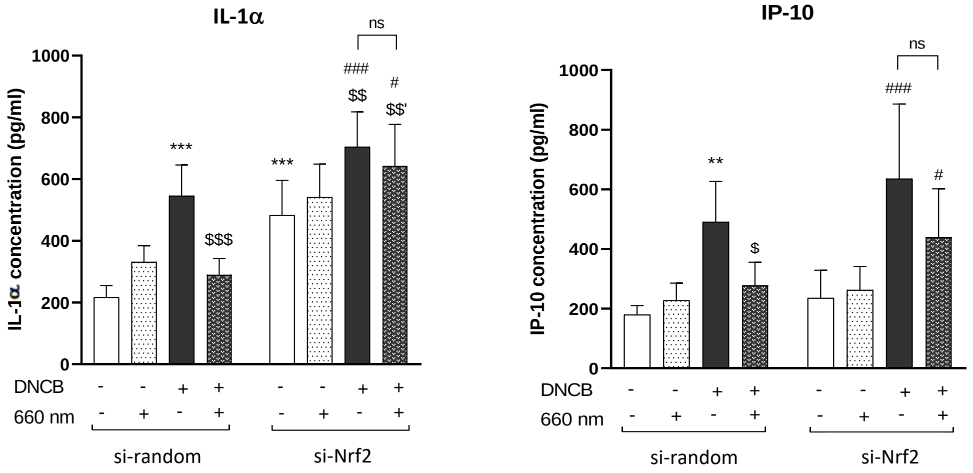

Figure S2.

IL-1α and IP-10 levels secreted by KCs knocked down for Nrf2 in response to DNCB and red light exposure. KCs were transfected for 48 h by siRNA directed against Nrf2 (si-Nrf2) or random siRNA (si-random). KCs were then treated with DNCB (25μM) and exposed to red light (660 nm, 3 J/cm^2). IL-1α and IP-10 pro-inflammatory cytokines level was determined by Meso Scaled Discovery technology in the supernatant of KCs after 6 h of exposure to DNCB with or without illumination. Reported data are mean ± SEM of 6 independent experiments. Two-Way ANOVA followed by Tukey post-hoc test, ** p < 0.01; *** p < 0.001 vs. untreated si-random; $ p < 0.05; $$ p < 0.01; $$$ p < 0.001 vs. DNCB-treated si-random group, $$′ p <0.01 vs. DNCB + 660 nm- treated si-random group; # p < 0.05, ### p < 0.001 vs. untreated si-Nrf2.

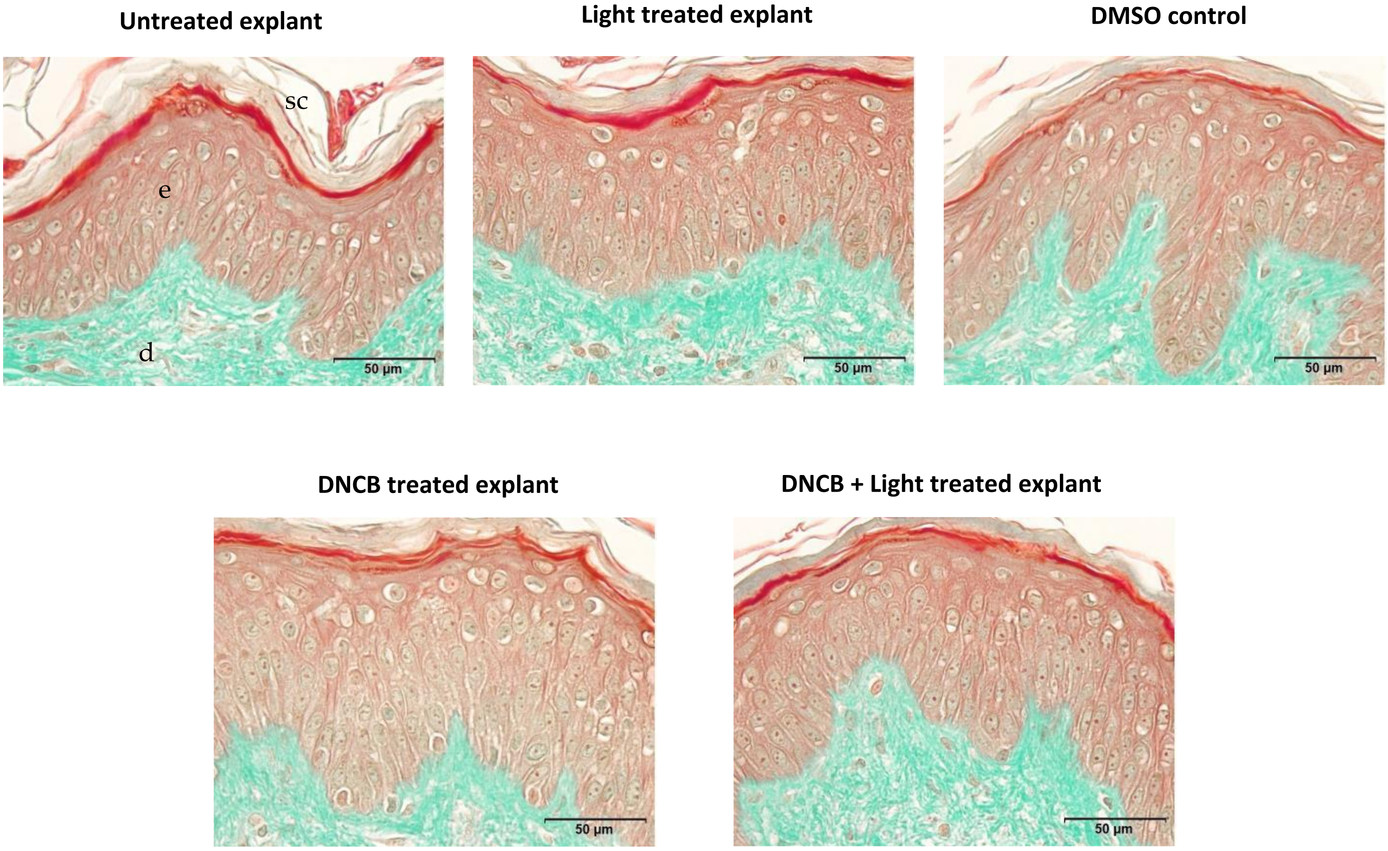

Figure S3.

Optical microscopy evaluation of the skin explants. Skin explants were treated with the excipient (DMSO 20%) or DNCB (0,25% *w/v*), with or without red light exposure (660 nm, 3 J/cm^2, 250 s). Histological control of the general morphology of the epidermal and dermal structures was assessed after 24 h of treatment by microscopical observation after Masson's trichrome (Goldner variant) staining of formol-fixed paraffin-embedded skin sections. Nuclei (dark brown), cytoplasm (Light greenish red), Keratin (bright red), collagen fibers (green). Images are representative of 3 independent explants from all groups. e, epidermis; d, dermis; sc, *stratum corneum.* Scale bars: 50 µm.

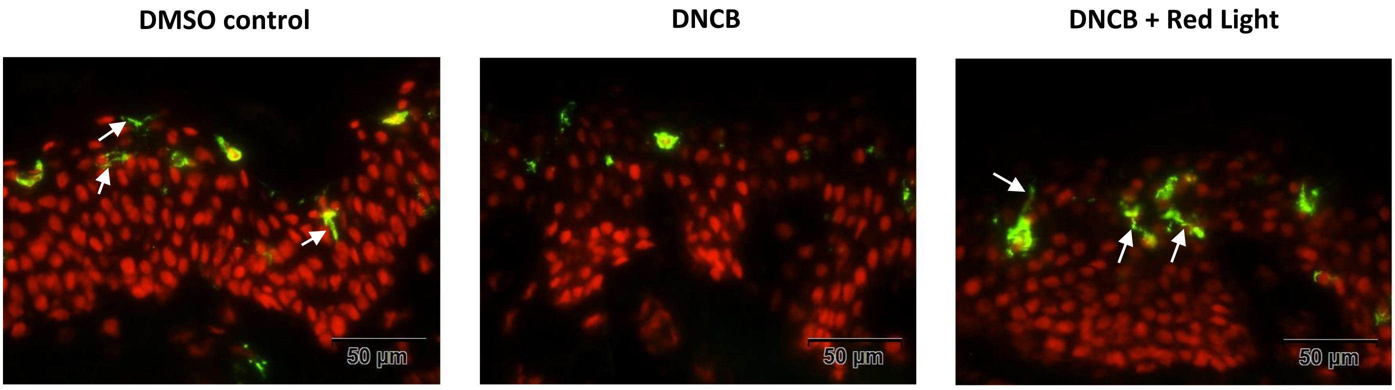

Figure S4.

Representative images of Langerhans cells' dendricity. Skin explants were treated for 24 h with the excipient (DMSO 20%), DNCB (0.25% *w/v*), with or without red light exposure (660 nm, 3 J/cm^2, 250 s). Langerin immunostaining was performed on frozen skin sections and revealed by AlexaFluor 488 (green). The nuclei were counterstained using propidium iodide (red). The white arrows point to the Langerhans cells' dendritic extensions.

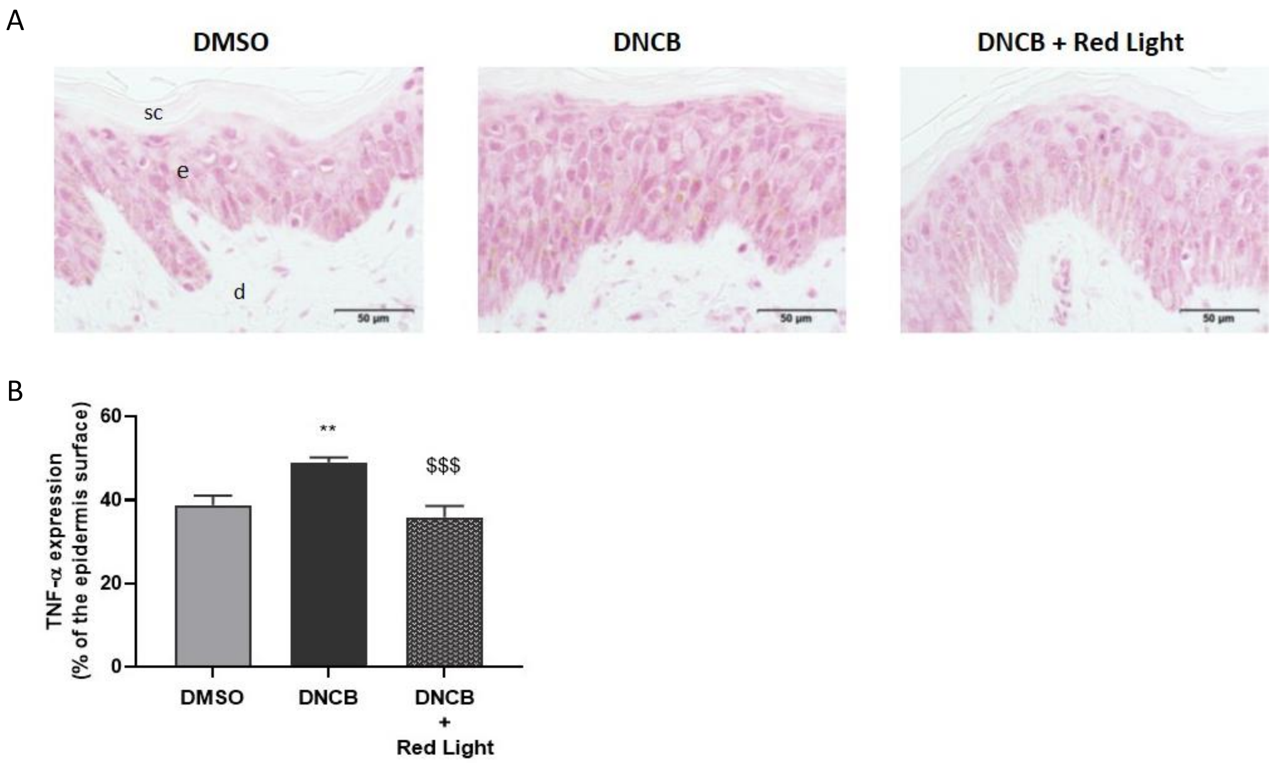

Figure S5.

Red light decreases DNCB-induced TNF-α expression in the epidermis. Human skin explants were treated topically with the excipient (DMSO 20%) or DNCB (0.25% *w/v*) with or without red light exposure (660 nm, 3 J/cm^2, 250 s). (A) TNF-α immunostaining was performed on formalin-fixed paraffin-embedded skin sections and revealed by VIP, a peroxidase substrate, giving a violet staining once oxidized. The pictures in this figure represent at least 9 images (3 images from 3 different explants for each group). e, epidermis; d, dermis; sc, stratum corneum. Scale bars: 50 µm. (B) Quantification of TNF-α expression in the epidermis assessed by image analysis and calculation of the surface percentage of the epidermis covered by the staining. Histogram represents the mean ± SEM of 9 different measures. ANOVA followed by Tukey post-hoc test, ** $p < 0.01$ vs. DMSO-treated control group; $$$ $p < 0.001$ vs. DNCB-treated group.

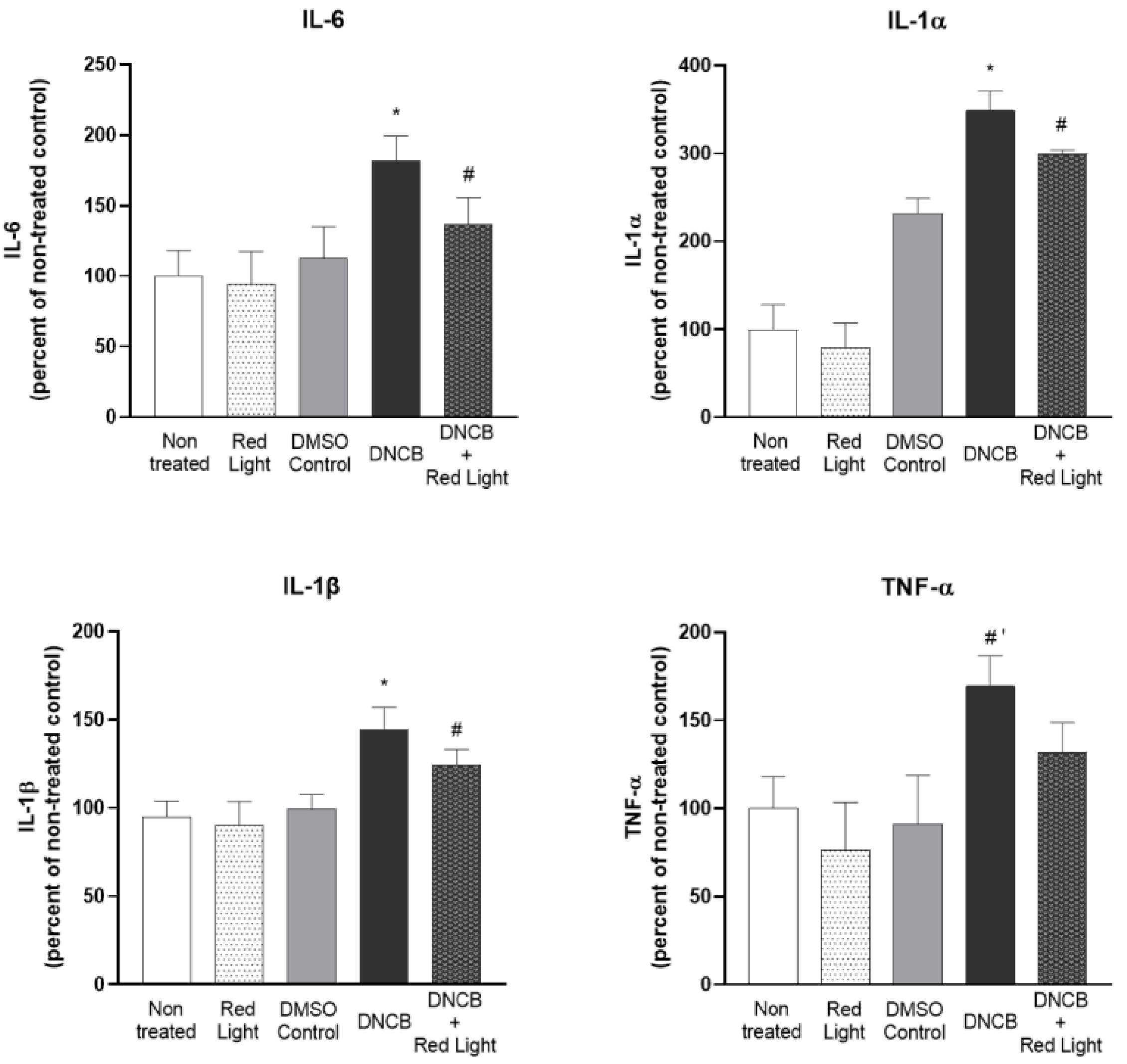

Figure S6.

Red light slightly decreased DNCB-induced pro-inflammatory cytokine secretion in the skin explants. Human skin explants were treated topically with the excipient (DMSO 20%) or DNCB (0.25% *w*/*v*) with or without red light exposure (660 nm, 3 J/cm^2, 250 s). Quantification of IL-6, IL-1α, IL-1β and TNF-α secretion level was assessed by Meso Scaled Discovery multiplex assay in the supernatant of the skin explants after 24 h of exposure to DNCB with or without red light illumination. Histogram represents the mean ± SEM of 3 independent measurements obtained from 3 different explants per group. Data were expressed as percentage of non-treated control group. One-way ANOVA followed by Holm-Sidak post-hoc test, * $p < 0.05$ vs. DMSO-treated group; # $p < 0.1$ vs. DNCB-treated group; #' $p < 0.1$ vs. DMSO-treated group.
